# Critical and Ictal Phases in Simulated EEG Signals on a Small-World Network

**DOI:** 10.3389/fncom.2020.583350

**Published:** 2021-01-08

**Authors:** Louis R. Nemzer, Gary D. Cravens, Robert M. Worth, Francis Motta, Andon Placzek, Victor Castro, Jennie Q. Lou

**Affiliations:** ^1^Department of Chemistry and Physics, Halmos College of Arts and Sciences, Nova Southeastern University, Fort Lauderdale, FL, United States; ^2^Department of Health Informatics, Dr. Kiran C. Patel College of Osteopathic Medicine, Nova Southeastern University, Fort Lauderdale, FL, United States; ^3^Department of Mathematical Sciences, Indiana University – Purdue University Indianapolis, Indianapolis, IN, United States; ^4^Department of Mathematical Sciences, Florida Atlantic University, Boca Raton, FL, United States; ^5^Department of Medical Education, Dr. Kiran C. Patel College of Allopathic Medicine, Nova Southeastern University, Fort Lauderdale, FL, United States; ^6^College of Medicine and Health Sciences, Khalifa University, Abu Dhabi, United Arab Emirates

**Keywords:** epilepsy, epileptic seizures, epileptogensis, small-world networks, simulation—computers, neuron, criticality, phase transition

## Abstract

Healthy brain function is marked by neuronal network dynamics at or near the critical phase, which separates regimes of instability and stasis. A failure to remain at this critical point can lead to neurological disorders such as epilepsy, which is associated with pathological synchronization of neuronal oscillations. Using full Hodgkin-Huxley (HH) simulations on a Small-World Network, we are able to generate synthetic electroencephalogram (EEG) signals with intervals corresponding to seizure (ictal) or non-seizure (interictal) states that can occur based on the hyperexcitability of the artificial neurons and the strength and topology of the synaptic connections between them. These interictal simulations can be further classified into scale-free critical phases and disjoint subcritical exponential phases. By changing the HH parameters, we can model seizures due to a variety of causes, including traumatic brain injury (TBI), congenital channelopathies, and idiopathic etiologies, as well as the effects of anticonvulsant drugs. The results of this work may be used to help identify parameters from actual patient EEG or electrocorticographic (ECoG) data associated with ictogenesis, as well as generating simulated data for training machine-learning seizure prediction algorithms.

## Introduction

The human brain must remain sensitive to new stimuli and coordinate spatially distant information processing modules to function optimally in a continuously changing environment. To accomplish this, the brain needs to dynamically operate at or near a critical state (Beggs and Timme, 2012). A failure to remain at this “edge of chaos” (Waldrop, 1992), a point poised between insensitivity and hyper-synchronization, can lead to neurological disorders, including epilepsy (Meisel et al., 2012).

A biological system, such as the human brain, in the critical state near a phase transition, is maximally sensitive to external influences (Larremore et al., 2011), and is thus most efficient in amplifying small perturbations. Such a state also allows for long-range coordination between brain regions, with a theoretical correlation length that diverges to infinity. At this intermediate critical phase, neuronal bursts exhibit power law statistics (Chialvo, 2010), with no typical spatial or temporal scale, unlike the seizure or subcritical phases. The properties of a system that are poised at the critical state of a phase transition may be very different than those of states on either side. In particular, the sensitivity of the system to external perturbations and information processing power is often maximized at this point. For example, in the case of magnetic susceptibility, at a critical temperature, flipping a single spin may lead to a cascade of magnetization changes that is not possible at either very low or very high temperatures. This “critical brain hypothesis” that links the physics of phase transitions with neuroscience was first introduced by Alan Turning seven decades ago, and has grown in acceptance due to increasing empirical and theoretical support (Turing, 1950). The theory has been questioned because of the apparent difficulty of satisfying the requirement for the parameters to be fine-tuned within a very tiny region of parameter space corresponding to the critical phase, as this would be very unlikely to occur by chance alone. However, recent research has demonstrated that the brain uses active homeostatic mechanisms (Beggs, 2019), including synaptic rewiring based on spike-timing dependent plasticity, to remain at the critical state (Shin and Kim, 2006; Ma et al., 2019).

## Background

Epilepsy is one of the most common central nervous system (CNS) diseases (Zack and Kobau, 2017), affecting ~50 million individuals worldwide, including both men and women of varying ages. It is a chronic neurological disorder characterized by a persisting predisposition to generate epileptic seizures and by the resultant neurobiological, cognitive, psychological, and social consequences. An epileptic seizure is a sudden, transient, and uncontrolled electrical disturbance in the brain. The signs and symptoms are caused by abnormal excessive or synchronous neuronal activity. Seizures can cause sudden changes in patient behavior, movements, feelings, or in levels of consciousness. Most patients have little or no warning before a seizure occurs, and this unpredictability can have profound impacts on in their lifestyle, including restrictions on driving, or constraints on employment opportunities.

Epilepsy research has enabled remarkable progress in broadening our understanding of the etiologies and mechanisms leading to epilepsy and its associated comorbidities. It has also brought interventions and treatments to improve the management of seizures and their comorbid conditions and consequences. The prognosis for medical seizure control is good, with over 70% of patients achieving remission. Meanwhile, 30% of individuals with epilepsy remain uncontrolled with increased risk of adverse events and lifestyle disruptions. As a result, over the last three decades, researchers have aimed to gain insight into the underlying mechanisms of epilepsy in these patients with uncontrolled symptoms, hoping to identify biomarkers of disease activity that would indicate an impending seizure and allow them to take protective action or perhaps initiate some therapeutic intervention (Mormann et al., 2007).

The critical brain hypothesis is one approach to understanding the onset and lack of control in these epilepsy patients. Epilepsy is believed to occur when the human brain is unable to dynamically operate in or near this critical state. According to this hypothesis, the intermediate critical phase, as opposed to the ictal or subcritical phases, exhibits scale-free phenomena—with no typical spatial or temporal scale. This behavior can be quantified using a power law functional form of the number of simultaneously firing neurons. This differs from the ictal state, which consists of “all-or-nothing” pathological synchronization, and the subcritical state that has only locally disjoint firing with no long-range coordination. Power-law dynamics are a necessary, but not sufficient, observation to conclude that a system is in its critical state. More stringent tests for the relationship between the scaling exponents for the spatial and temporal sizes of bursts have been developed to distinguish actual critical behavior from other phenomena that can also give rise to power laws (Friedman et al., 2012).

To better understand this phenomenon, we employed the Hodgkin-Huxley equations (Hodgkin and Andrew, 1952) for modeling the dynamics of neurons. The model is computationally intensive, and as such, has usually been limited to simulating small networks of neurons for short time periods. Even with these constraints, distinct phases can be distinguished by plotting a histogram of the number of simultaneously firing neurons for each simulation time step, with the ictal phase associated with significant deviations from power-law behavior. This is particularly true when the network topology is chosen to be Small World (SW) (Humphries and Gurney, 2008). SW networks have many local connections between nearby neurons, and a few long-range bridges. It is widely believed that the human brain possesses many aspects of SW architecture (Bassett and Bullmore, 2006). This allows for the modularity of high clustering coefficients to coexist with the rapid and efficient communication of short path lengths. In the case of focal epilepsy, it is possible that these adaptations become harmful. A seizure focus is thought to recruit connected neurons into a growing synchronized cluster. This process nucleates uncontrolled growth that can rapidly spread on SW networks (Hong et al., 2002). The natural refractory period inherent in neural spiking normally protects the brain from reaching this synchronized ictal phase. However, when connectome plasticity increases the synaptic weights between neurons—either because of trauma, the reinforcement of neural pathways from previous seizures, or channelopathies that lengthen the action potential—these protections can fail.

Therefore, our research efforts focus on access to high-resolution data, in this case, the simulated voltages of individual neurons, in contrast with patient measurements that only capture averaged field potentials at best. This allows us to easily classify the phase—silent, exponential, power, or ictal—to better understand the process of ictogenesis. Using full HH simulations on an SW network, we have generated synthetic EEG signals with intervals corresponding to seizure (ictal) or non-seizure (interictal or subcritical) states that can occur based on the hyperexcitability of the artificial neurons and the strength and topology of the synaptic connections between them. By generating this synthetic data, we aim to train machine learning (ML) algorithms for predicting seizures in patients with epilepsy. Due to the difficulty in acquiring high-quality patient data, and the relative rarity of seizures events compared with interictal intervals, the ability to generate synthetic data may alleviate an important bottleneck in seizure prediction methods (Aznan et al., 2019). Rapid advances in computational power permit more realistic full HH simulations that were considered not practical only a few years ago. Overall, the goals here are twofold. First, to better understand the causal biophysical abnormalities of the ictal transition. Second, to generate a set of surrogate EEG data for use as input to ML algorithms which can then leverage their power to identify this interval and rigorously characterize the spatiotemporal characteristics. Since the biophysical changes generating the surrogate data are known, it should be helpful in “reverse engineering” the ML results to better understand possible seizure prediction strategies.

## Method

HH neurons in a SW network have intrinsic regulatory features, including threshold-and-fire activity and refractory periods. As a result, they are comparable with the archetypal self-organized criticality (SOC) (Rubinov et al., 2011) situations of sandpile avalanches and earthquakes. In each of these cases, SOC is a natural consequence of the underlying balance of slow and fast variables corresponding to loading and release, respectively (Dickman et al., 1998, 2000; Gal and Marom, 2013).

Computational models of isolated HH neurons show that criticality requires exquisite fine-tuning (Chua, 2013) of processes, like the slow inactivation of sodium channels, in order for the cell to remain excitable (Ori et al., 2018) but not oscillatory. Based on the theory of percolation phase transitions (Breskin et al., 2006; Zhou et al., 2015), in which adjacent nodes of a network are connected with probability *p*, the number distribution*, n*, of clusters of size *s*, obeys the proportion:

$$\begin{array}{l} n\left(s,p\right)\;\alpha \;s^{-\tau }e^{-s/s_{0}} \end{array}$$

Where *s*_0_ is a function of p. For values of p below the critical percolation threshold p_c_, there is a typical cluster size s_0_. The formation of larger clusters is strongly suppressed, since the exponential function dominates the power law except for very low values of *s*. However, at the critical threshold, the value of s_0_ diverges to infinity. This means that there is no longer a “typical” cluster size—the relation has become scale free—resulting in a power-law distribution with many small clusters, fewer medium clusters, and a small number of large clusters:

$$\begin{array}{l} n\left(s,p=p_{c}\right)\;\alpha \;s^{-\tau } \end{array}$$

Extracted burst sizes show a peak around the physiological value of the Na^+^ inactivation ion-channel gating parameter. Coordination both within and between specific brain modules is thought to be maximized at or near the critical state, since activity at all length scales becomes important. This is not possible in the hypercritical (Netoff et al., 2004) ictal state seen in epilepsy, when global synchronization overwhelms everything else.

To simulate the dynamics of the small-world neuron networks, we introduce the “Theoretical HH Ion-Gated Network Connectome Electroencephalographic Replicator” (THINKER) 1.0 and 2.0 mathematical models. THINKER version 1.0 simulations were instantiated in *Mathematica*. First, the Watts-Strogatz algorithm (Watts and Strogatz, 1998) was used to generate the SW directed-network architecture. The connectivity matrix and directionality were fixed for all runs, but the weights could vary with uniform probability from 0 to g_max_. The HH coupled differential equations we implemented for each neuron using Euler's method in 0.1 ms time steps to find the voltage *V* are:

$$\begin{array}{l} -C\frac{dV}{dt}=m^{3}hg_{Na}\left(V-V_{Na}\right)+n^{4}g_{K}\left(V-V_{K}\right)\\ \;+g_{leak}\left(V-V_{leak}\right)+I_{inject} \end{array}$$

Where m, n, and h, are the voltage-dependent gating parameters for Na^+^ activation, K^+^ activation, and Na^+^ inactivation, respectively. The term I_inject_ represents incoming synaptic stimuli from connected neurons, and C is the membrane capacitance (Table 1). The response of these parameters to changes in voltage are controlled by the opening (α) and closing (β) rate-constants for individual gates in the neuron's membrane:

$$\begin{array}{l} \frac{dm}{dt}=\alpha_{m}\left(1-m\right)-\beta_{m}m\\ \frac{dn}{dt}=\alpha_{n}\left(1-n\right)-\beta_{n}n\\ \frac{dh}{dt}=\alpha_{h}\left(1-h\right)-\beta_{h}h \end{array}$$

This implies the steady state values for z = {m,n,h} are:

$$\begin{array}{l} z_{\infty }=\frac{\alpha_{z}}{\alpha_{z}+\beta_{z}} \end{array}$$

with corresponding rate constants, in inverse seconds, assuming constant voltage:

$$\begin{array}{l} k_{z}=\frac{1}{\alpha_{z}+\beta_{z}}\;=1/\tau_{z} \end{array}$$

The “stickiness” of sodium inactivation gates can be modeled by multiplying α_h_ and β_h_ by the same constant s:

$$\begin{array}{l} \alpha_{h}^{\prime }=s\alpha_{h}\\ \beta_{h}^{\prime }=s\beta_{h} \end{array}$$

This decreases the rate constant for h, which is the variable describing the gates that inactivate the sodium channels when the neuron is supposed to return to its resting state after firing. The result is an elongation of the characteristic time for h to equilibrate to changes in voltage, from τ_h_ to sτ_h_, while keeping the steady state value of h unchanged.

**Table 1:** Model parameters for the THINKER 1.0 Simulation.

**Parameter**	**Symbol**	**Value**
Normalized membrane capacitance	C	1
Maximum Na^+^ conductivity	g_Na_	20
Maximum K^+^ conductivity	g_K_	12
Maximum leak conductivity	g_Leak_	0.05
Na^+^ reversal voltage	V_Na_	50 mV
K^+^ reversal voltage	V_K_	−90 mV
Leak reversal voltage	V_Leak_	−60 mV
Na^+^ deactivation “stickiness”	S	*Varies*
Minimum connectivity weight	g_min_	0
Maximum connectivity weight	g_max_	*Varies*
Initial voltage	V_0_	−64 mV
Time step	dt	0.1 ms

The explicit forms of the α and β parameters for neuron voltages in millivolts are given by:

$$\begin{array}{l} \alpha_{m}\left(V\right)=0.1\frac{V+40}{1-Exp\left[-0.1\left(V+40\right)\right]}\\ \beta_{m}\left(V\right)=4\;Exp\left[-0.05\left(V+65\right)\right]\\ \alpha_{n}\left(V\right)=-0.01\frac{V+55}{Exp\left[-0.1\left(V+55\right)\right]-1}\\ \beta_{n}\left(V\right)=0.25\;Exp\left[-0.0125\left(V+65\right)\right]\\ \alpha_{h}\left(V\right)=s\;0.07\;Exp\left[-0.05\left(V+65\right)\right]\\ \beta_{h}\left(V\right)=\frac{s}{Exp\left[-0.1\left(V+35\right)\right]+1} \end{array}$$

A hyperbolic tangent is used for the transfer function for synaptic connections. One neuron is “voltage clamped” to a white-noise source that drives the system without imparting a characteristic frequency. THINKER 2.0 is written in *Python* and follows Ermentrout and Terman (Ermentrout and Terman, 2010) with the same structure but slightly different parameters. The simulation outputs the voltages of each simulated neuron at every time step. The data are converted into a raster array that records the firing of each neuron when its voltage exceeds a preset threshold. The phases can be labeled by fitting a histogram of simultaneously firing neurons, to either an exponential or power-law function, following the established methods for identifying critical behavior. These neuronal-level, voltage-resolved data are generally not available to the ML algorithm, which would as a matter of practice only have access to EEG or electrocorticographic (ECoG) data.

## Results

**Figure 1:**
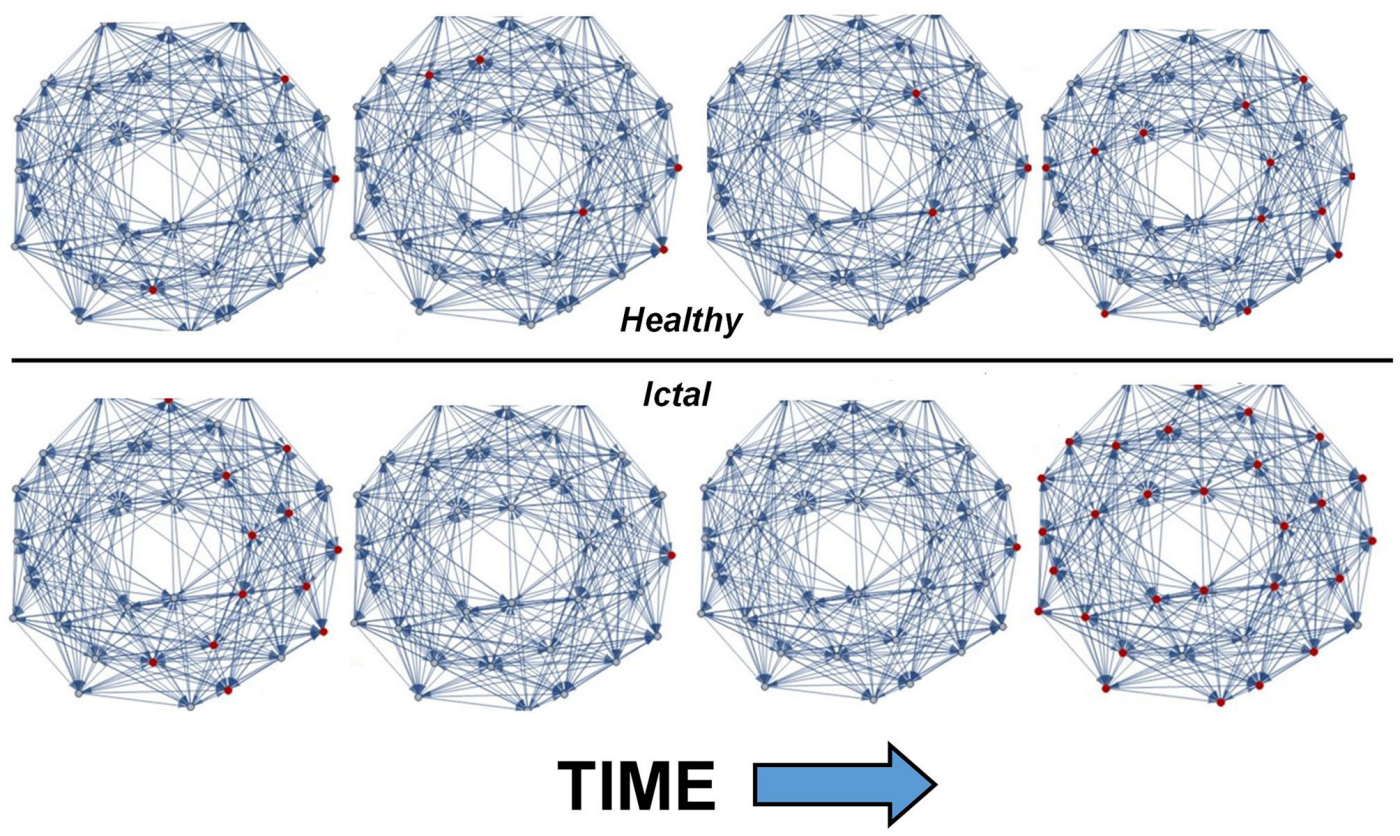
Visual representation of THINKER 1.0 simulation time evolution showing healthy (top) and ictal (bottom) phases. The synaptic connections between neurons are shown in blue, and the firing neurons are marked red. Time increases to the right, and the rightmost neuron is “voltage-clamped” to a white noise source. In the healthy/critical phase, the activity follows a power-law distribution, so “bursts” of all sizes are possible. By contrast, in the ictal/supercritical phase, “all-or-nothing” pathological synchronization is observed.

**Figure 2:**
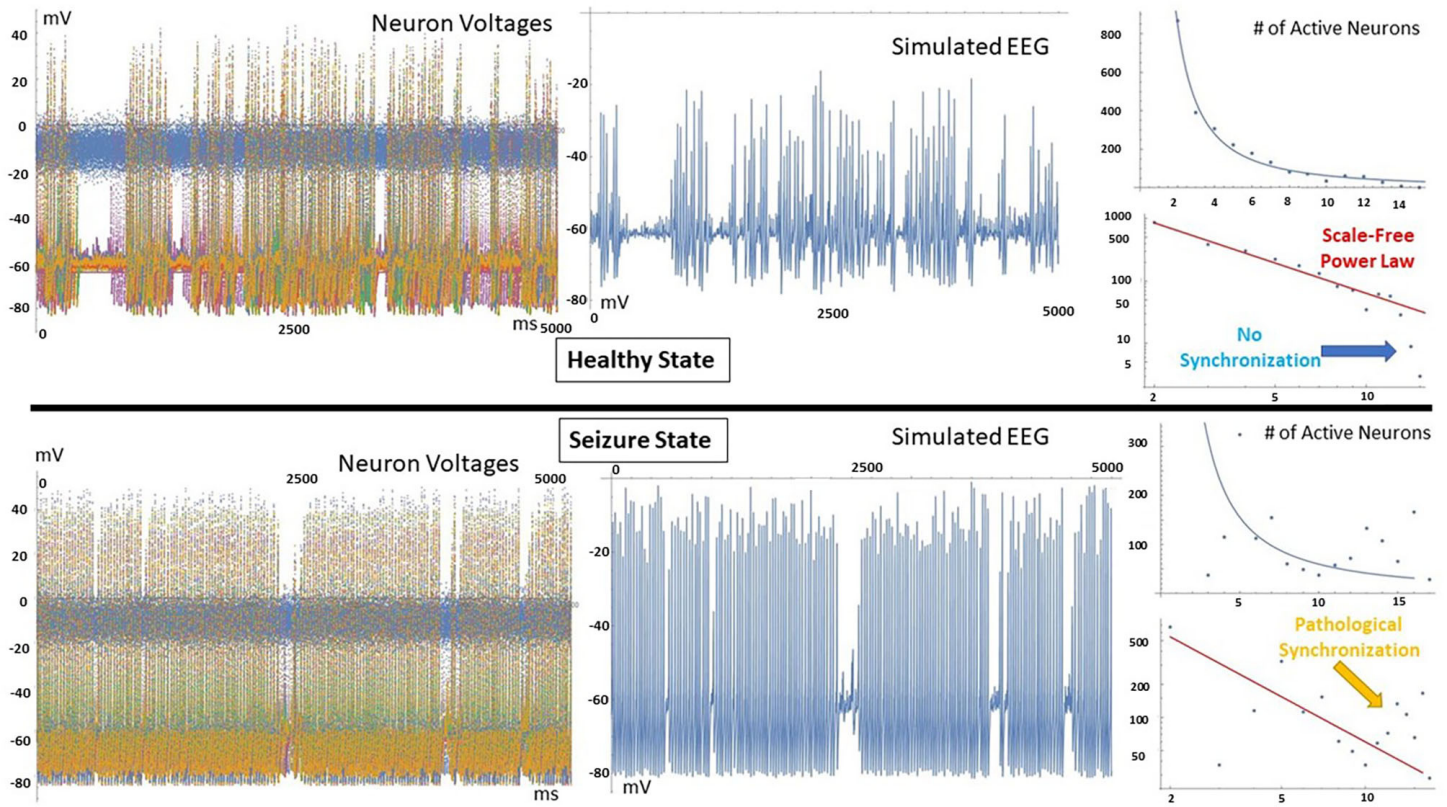
(Left) All simulated neuron voltages. The blue trace is the voltage-clamped neuron driven by white noise. (Middle) Resulting synthetic EEG, calculated at each time step as the mean value of all neurons. (Right) Corresponding histogram showing the number of time steps that have that number of simultaneously firing neurons. In the healthy state, a power-law relationship is observed, and time steps that show near complete synchronization are very rare. In contrast, the seizure state has a spike at high node numbers, meaning that pathological synchronization occurs.

Figure 1 shows the output of THINKER 1.0 simulations on small-world networks with the firing neurons labeled in red. In the healthy phase (top), the activity has bursts with a distribution of sizes, with coordination on local and extended length scales. In contrast, the supercritical ictal state (bottom) has globally synchronized firing with timesteps that have almost all the nodes either on or off simultaneously. In Figure 2, the difference between these phases can also be demonstrated using the simulated EEG. The left panels for the healthy and seizure state show the individual neuron voltages, and the middle graphs display the overall mean voltage at each time step. These are called “simulated” or “synthetic” EEGs, although they can be compared with either conventional scalp electrocochleographic (EEG) or intracranial electrocorticographic (ECoG) patient data. The right panels show the calculated histograms of the number of simultaneously firing neurons. The upper subfigure is linear, while the lower subfigure is log-log scale so that power laws will be represented by straight lines. In the healthy critical phase, the histogram follows a power-law form, and the occurrence of more than 10 simultaneously firing neurons is suppressed. By contrast, in the ictal seizure state, the histogram has a spike around 12 simultaneous neurons, corresponding to near complete synchronization.

**Figure 3:**
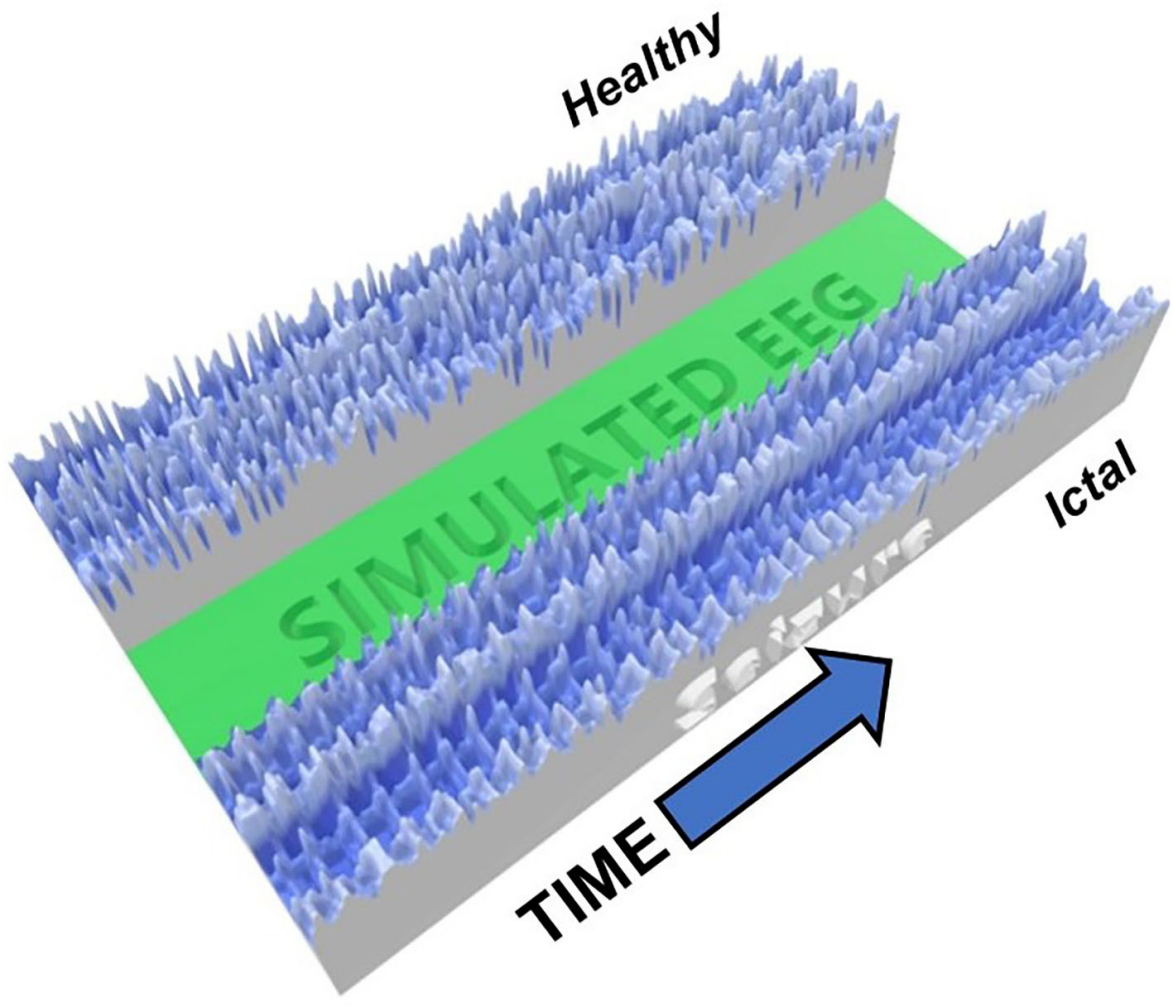
Image of 3D-Printed model showing an example of critical (healthy, top) and ictal (seizure, bottom) phases. The heights represent the neuron voltage for each time step. The synchronization in the seizure state is readily visible, while the signals in the healthy state are much more varied.

To further visualize the different brain phases, an image of a 3D-printiable file is shown in Figure 3. Here, the voltages at each time times for all neurons are represented by the height of the model. The difference between the complexity of the critical healthy state and the repetitive synchronization of the ictal state can be observed.

**Figure 4:**
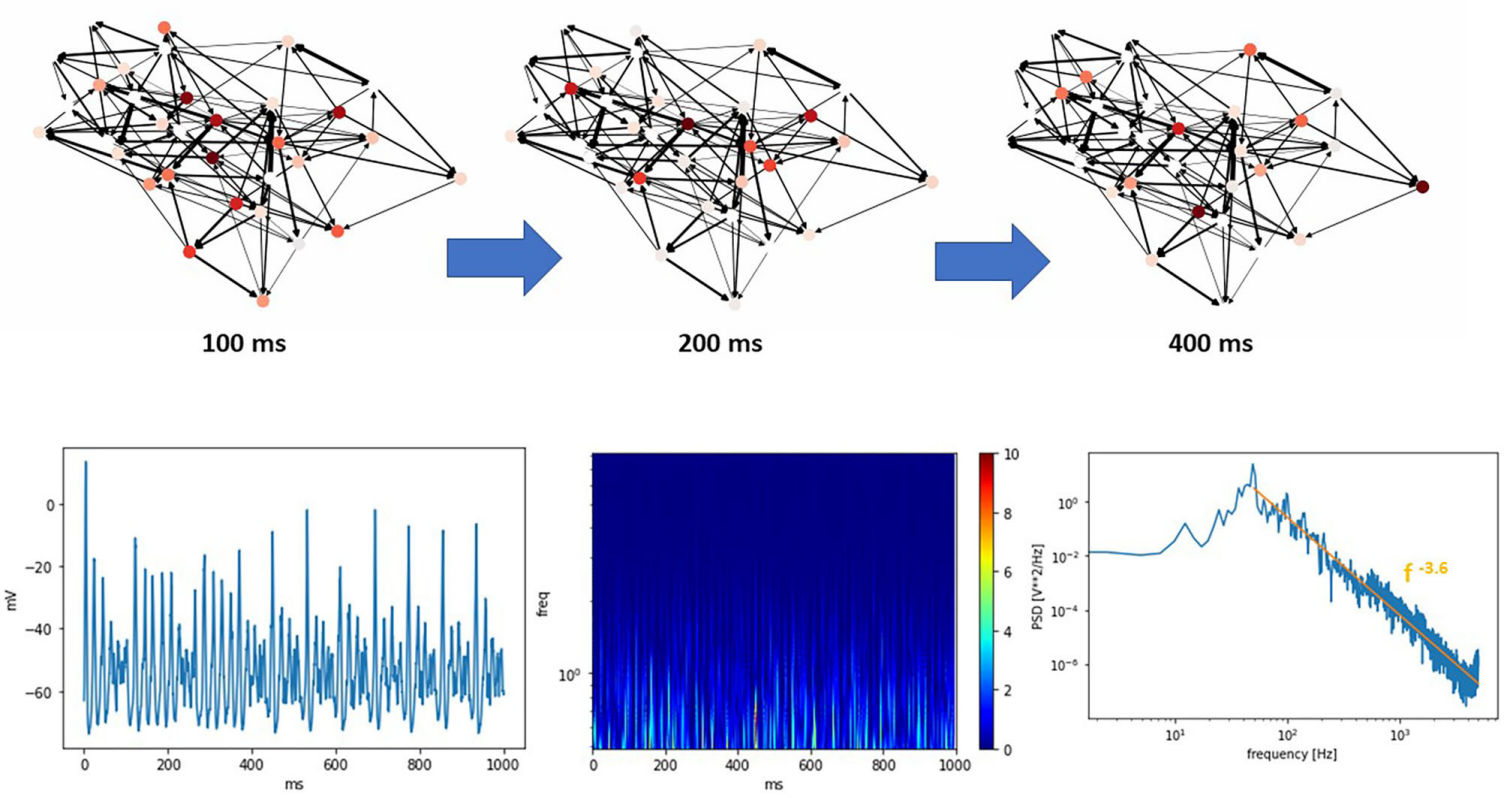
(Top) A THINKER 2.0 Small World network showing critical phase behavior over time. Local and global coordination between bursts is seen, as observed in other systems with SW topologies. An animated movie version is available in the Supplementary Material. (Bottom) The corresponding simulated EEG, scalogram, and power spectral density plots. In the power spectral density plot, the data at high frequencies closely follow a power law with exponent −3.6 (orange line).

Figure 4 (top) shows a THINKER 2.0 small world network near the critical state at different time points. An animated movie version is available as part of the Supplementary Material. Below are the corresponding synthetic EEG, wavelet transform scalogram, and power spectral density.

The wavelet transform is accomplished by convolving a set of orthogonal wavelet “chirps” that have identical shape, but different scaling factors, with the data (Akansu et al., 2010). The scalograms produced are similar to the spectrograms created using short-time Fourier transforms. The primary difference is the dynamic way in which the inherent tradeoff between temporal and frequency resolution is handled. The wavelet transform uses basis functions that are localized in both time and frequency. As a result, at low frequencies scalograms possess good frequency resolution at the expense of poor temporal resolution. Conversely, high frequencies enjoy good time resolution but reduced frequency resolution. Wavelet transforms reveal the complexity of activity present in actual human brain function, particularly in the high frequency regions. These can be interpreted as non-periodic neuronal bursts of varying sizes within these frequency bands, as observed with normal brain function. In the high frequency region of the power spectral density, the data closely follows a power law with exponent -3.6, as marked by the orange line. This agrees with previous measurements of human brain activity (Miller et al., 2009).

**Figure 5:**
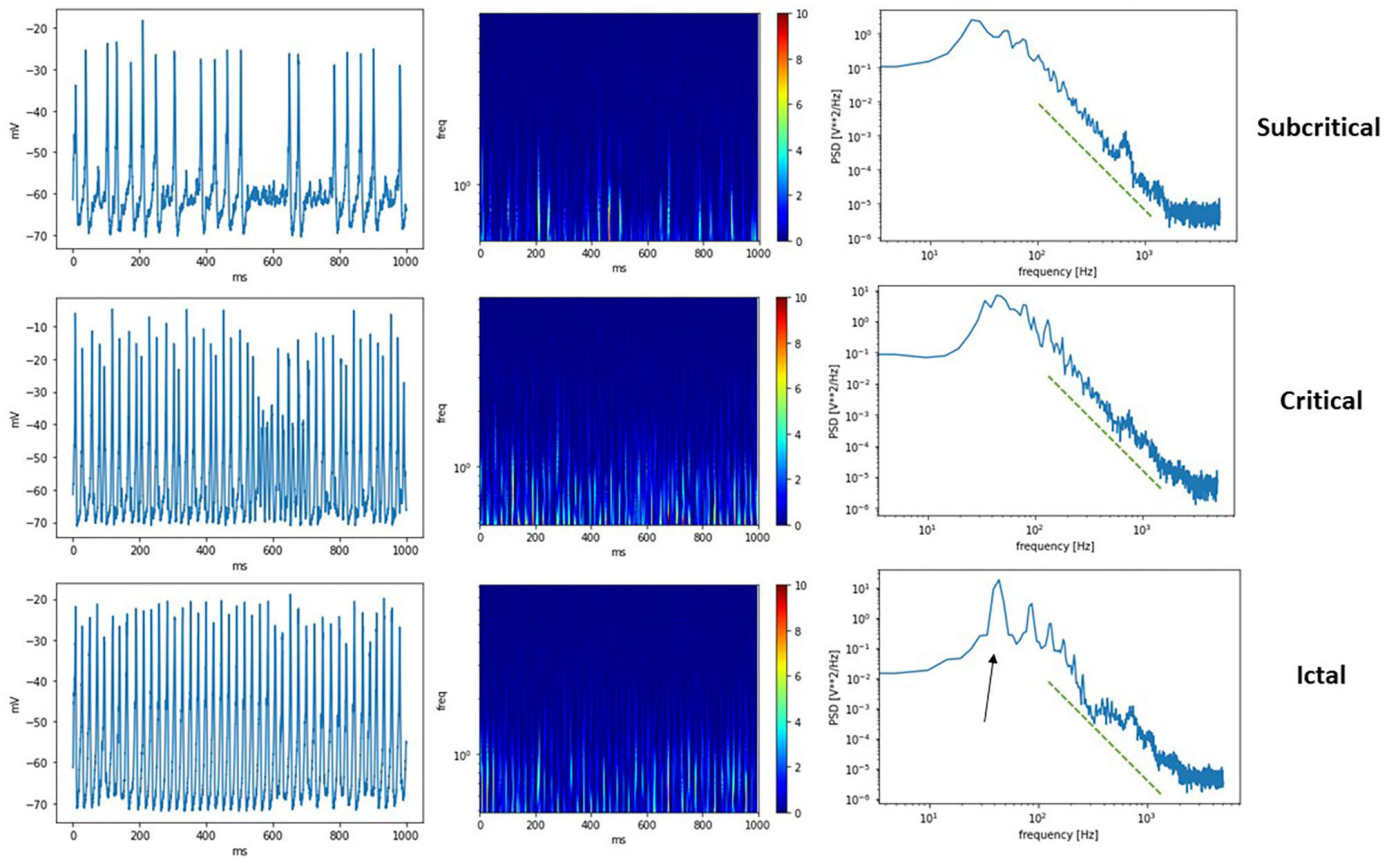
(Left) Synthetic EEG from a subcritical, critical, a supercritical ictal state. (Middle) Corresponding wavelet transform scalograms. (Right) Calculated power spectral density plots. The green dashed line shows the same power law as a guide for the eye. The critical state follows the line most closely, while the ictal state has peaks representing synchronization at that frequency (black arow).

A comparison of the different phases possible from THINKER 2.0 simulations is shown in Figure 5, where the synthetic EEG, corresponding wavelet transform, and power spectral density are shown for the subcritical, critical, and ictal regimes. Again, the critical phase shows the most complex activity in the wavelet transform, and most closely follows a power law (green line) in the power spectral density plot. The ictal state has a prominent spike (black arrow) representing synchronization.

**Figure 6:**
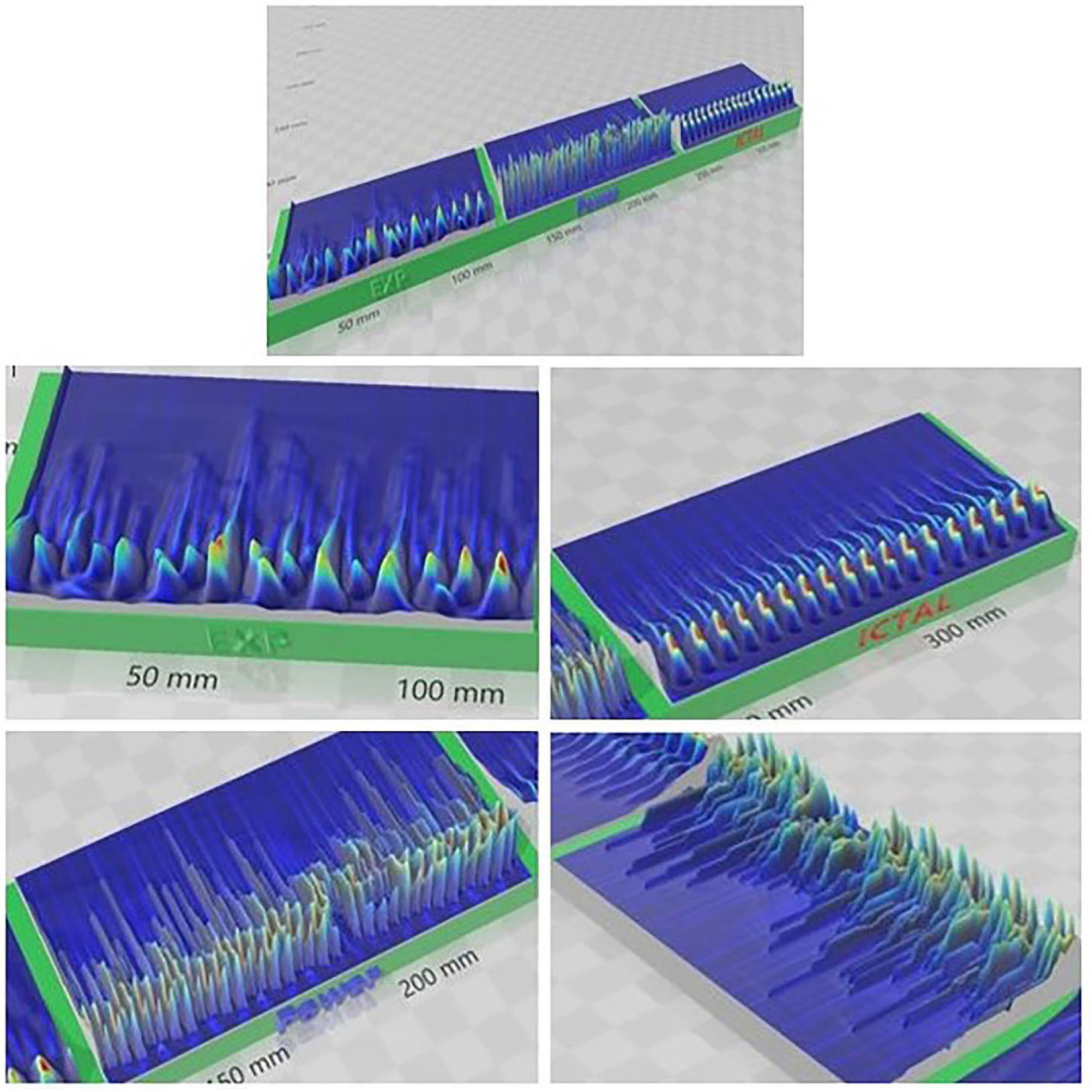
3D-Printed representation of subcritical (EXP) critical (Power) and supercritical (Ictal) phases. Complex activity is evident at higher frequencies most in the critical phase. Compare this with the repetitive nature of the ictal phase and the reduced activity in the subcritical phase.

The differences between the subcritical/interictal, critical, and ictal phases are easy to see when the scalograms are converted into a 3D-printed representation (Figure 6). The subcritical phase (“EXP” for exponential) has too little activity overall, while the ictal phase is highly locked into a single pattern. Only the critical phase (“Power” for power law) has the complexity, in both the low and high frequency bands, to capture the neurocorrelates of healthy cognition.

**Figure 7:**
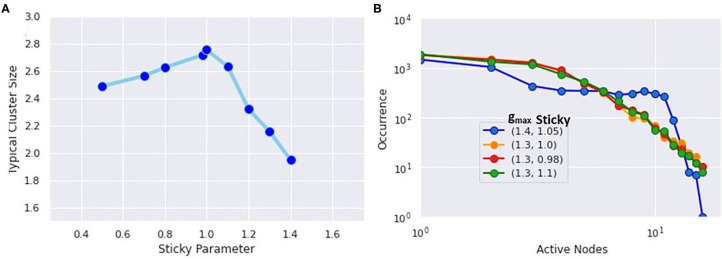
**(A)** Extracted mean cluster size using the formula from percolation theory. The largest value occurs near the physiological value of the Na^+^ inactivation parameter called “sticky.” **(B)** Histogram of simultaneously firing neurons. The critical state most closely follows a power-law distribution, while the subcritical states show exponential decay.

The uniqueness of the critical state also appears in the histograms of simultaneously firing neurons as seen in Figure 7. Here, the network topology was frozen, and only the stickiness was varied. The extracted values of the mean cluster sizes are shown in Figure 7A. As predicted by percolation theory, subcritical states will have large clusters exponentially suppressed, while the mean cluster size diverges in the critical state, leaving a scale-free power-law relationship. Here, the g_max_ represents the maximum synaptic weight of connected neurons, and the “sticky” parameter again controls the Na^+^ channel inactivation after each neuron fires. The finding that the critical state occurs with a “sticky” value of 1.05, with 1.0 corresponding to the physiological value, agrees with the concept that the brain is regulated to be at or slightly below the critical threshold (Beggs, 2018). In the histograms of simultaneously firing neurons shown in Figure 7B, a power law will produce a straight line, while an exponential function will curve downward. Only the histogram for the critical state exhibits a power law tail.

**Figure 8:**
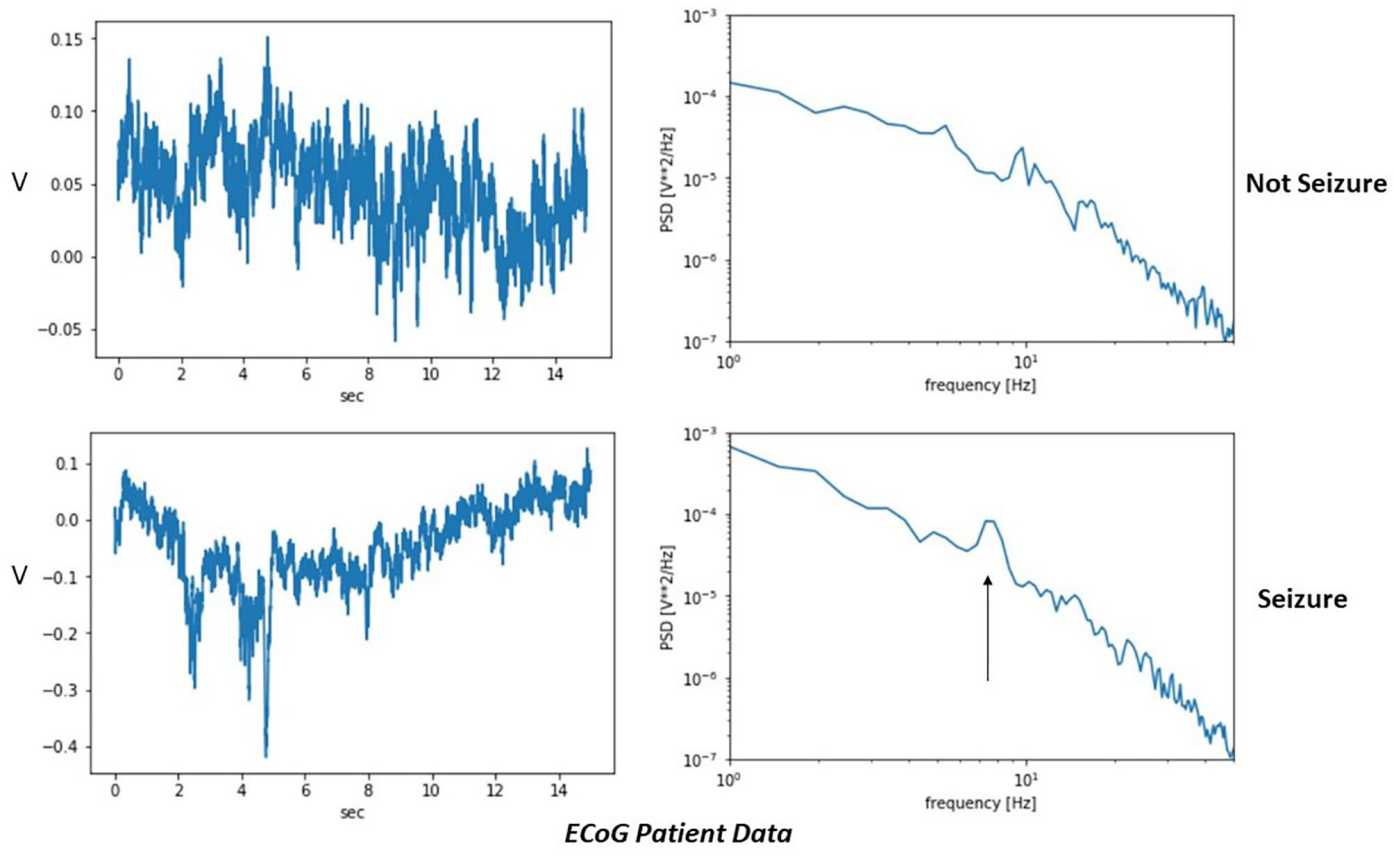
Example high-resolution patient data using electrocorticography (ECoG) intracranial electrodes. A time interval marked by the clinician as a seizure has a peak in the power spectral density (black arrow) that is absent during the non-seizure sample.

Figure 8 shows actual ECoG patient data collected from intracranial electrodes. The readings were taken as part of preoperative testing prior to epilepsy surgery. A clinician marked time intervals as either interictal (not seizure) or ictal (seizure). Here, a peak in the power spectral density around 8 Hz is seen in the ictal but not interictal data. The shift in peak frequency compared with the simulations may reflect differences in the natural oscillation periods for the modeled network using the chosen parameters.

## Discussion

Understanding ictogenesis, the cascade of biophysical events that culminates in a clinically expressed epileptic seizure, has remained an elusive goal, of special concern for those epilepsy patients whose seizures remain uncontrolled with currently available drugs and approaches. While mechanisms of disease activity have been proposed and most seizures remain well-controlled, a significant subset of patients remain at high risk for adverse events and require lifestyle adjustment that negatively impacts their ability to perform activities of daily living. It is thought that epilepsy occurs when the human brain is unable to dynamically operate in or near this critical state, where certain biological neuronal networks work near phases of pathological synchronization and insensitivity. A failure to remain at this point poised between instability and homeostasis can lead to epilepsy. One of the most prominent approaches to understanding the underlying mechanisms of epileptic seizure activity has been mathematical modeling of the relevant physiological processes. The formalism described by Hodgkin and Huxley in 1952 provides an elegant mathematical description of neuronal behavior and is considered the gold standard for describing neuronal physiology. However, the computational complexity involved in modeling systems of neurons large enough to exhibit meaningful behavior have up until now rendered models of this type computationally intractable. As a result, previous such approaches have generally worked with some sort of approximated forms, the assumptions of which may vitiate some of the conclusions drawn from such models. Meanwhile, advances in computational power have now made it feasible to perform simulations using full HH models describing a biophysically relevant number of neurons. In this paper, we described THINKER 1.0 and THINKER 2.0, which implement full HH model simulations with the neurons configured in a SW topology. It goes without saying that no simulation, especially with orders of magnitude fewer nodes, can reflect all of the complexity of the human brain, and we have focused on aspects more relevant for future machine learning algorithms for predicting seizures in patients with epilepsy. Here, the simulated networks reproduce both the power-law tail at high frequencies seen in the power spectral density of patient data at times of normal cognition, as well as the peaks corresponding to pathological synchronization that occur during seizures.

The work to date has yielded some interesting insights into the process of ictogenesis. The results of this modeling work identified three brain states—subcritical/interictal (non-seizure state, with little or no activity), critical (non-seizure state, with the number of active neurons characterized by a power law distribution), and supercritical (ictal seizure state, displaying pathological synchronization of large numbers of neurons). The model biophysical parameter whose variation determines in which state the system exists is *h* in the HH formalism, which describes the process of Na^+^ inactivation. On an individual neuronal basis, alteration of this parameter will affect the rate of neuronal repolarization, which in turn alters the spike frequency relative to other neurons, which can affect neuronal synchronization. In the model, we see an example where a single parameter can determine whether the system is in a normal critical state or transitions into a pathologic ictal regime. Future work will involve increasing the number of neurons, extending the simulation time, and looking at the effect of the other gating parameters on neuronal state.

One of the traditional criticisms of ML, including deep learning neural networks (DLNNs), has been that the trained systems appear to be “black boxes” for which it is not easy to understand how they are making decisions. The recent development of topological data analysis (TDA) (Carlsson, 2009) has led to attempts to treat the parameters of a trained DLNN system as just another data set. Then TDA methods can be applied to the data set of system weights to gain insights into how the DLNN system is making decisions. Such insights may ultimately lead to neurophysiologic insights into ictogenesis. ECoG data from epilepsy patients can serve as the foundation for refining and validating the THINKER models described in this article. This model-generated data can then be used as input to ML routines to attempt to identify biomarkers of impending seizures, and also to attempt to gain insight into the underlying neurobiology of ictogenesis. For ethical reasons, human data acquisition must be driven by clinical parameters and hence is available in extremely limited amounts. Once the simulated ECoG generated by the THINKER models has been validated against actual patient data, it can then serve as input to ML techniques to attempt further characterize electrophysiologic biomarkers of impending seizures. Although there are numerous ML methods, we anticipate using DLNNs in our initial work. DLNN has been found to generate increasingly abstract concepts as it progresses deeper into the hierarchy of hidden layers (Schmidhuber, 2015). This should increase the likelihood that the DLNN model can be useful for developing a system for predicting the real-time risk of impending seizures.

## Conclusion

The present work utilized full Hodgkin-Huxley simulations with artificial neurons connected in a small-world topology to demonstrate that alteration in a single neuronal parameter can catalyze the transition to the pathological synchronization characteristic of epilepsy, a change from non-seizure (interictal or subcritical) to seizure (ictal) states. These simulations help provide a better understanding of the ways network topology, synaptic strength, and neuron excitability influence both healthy brain function and pathological states such as epilepsy. A primary biomarker of the seizure state is a spike in either the power spectral density of an averaged signal like patient ECoG measurements, or the histogram of the number of simultaneously firing neurons in the highly spatially resolved simulation data. In addition, during the healthy intervals marked by activity at or near the critical point, the simulations reproduce power-law behavior seen in patient data. This project also makes more visible the distinct brain phases observed in actual patients. The next steps are obtaining ECoG recordings from patients with temporal lobe epilepsy who underwent presurgical evaluation for temporal lobectomy. This will allow us to refine and validate the insights from the models, especially important given the scarcity of actual patient ECoG data. The synthetic data generated by the model can also be used to train future machine-learning algorithms to assess the real-time risk of seizure onset.

## Data Availability Statement

The raw data supporting the conclusions of this article will be made available by the authors, without undue reservation.

## Author Contributions

LN created and performed the simulations and wrote portions of the manuscript. GC conceived the project and wrote portions of the manuscript. RW worked on the neuroscience aspects and wrote portions of the manuscript. FM and VC worked on the mathematical theory. AP worked on the physiological aspects and edited the manuscript. JL cowrote the introduction and edited the manuscript. All authors contributed to the article and approved the submitted version.

## Conflict of Interest

The authors declare that the research was conducted in the absence of any commercial or financial relationships that could be construed as a potential conflict of interest.
